# Progression of histological lesions after ABO incompatible kidney transplantation

**DOI:** 10.3389/fimmu.2022.969998

**Published:** 2022-10-06

**Authors:** Pierre Guy, Audrey Delas, Laure Esposito, Olivier Cointault, Magali Colombat, Nicolas Congy-Jolivet, Marc Raynaud, Nassim Kamar, Arnaud Del Bello

**Affiliations:** ^1^Department of Nephrology and Organ Transplantation, Centre Hospitalier et Universitaire (CHU), Toulouse, France; ^2^Department of Pathology, Toulouse University Hospital, Toulouse, France; ^3^Centre Hospitalier et Universitaire, Université Paul Sabatier Toulouse III, Toulouse, France; ^4^Laboratory of Immunology, Biology Department, Centre Hospitalier et Universitaire (CHU) de Toulouse, Toulouse, France; ^5^Paris Translational Research Epidemiology and Biostatistics Department, Université de Paris, INSERM U970, PARCC, Paris, France; ^6^Toulouse Institute for Infectious and Inflammatory Diseases (Infinity), INSERM UMR1043-CNRS 5282, Toulouse, France

**Keywords:** ABOi, ABO incompatible, histological evaluation, long - term effect, rejection, chronic antibody-mediated rejection (cABMR)

## Abstract

Recent large meta-analyses suggested a poorer long-term patients’ and grafts’ outcomes after ABO incompatible (ABOi) living-donor kidney transplantation (LDKT) compared to ABO compatible LDKT. However, little is known about the long-term histological pattern after ABOi LDKT. We compared the histological features observed on protocol biopsies from 03/11 to 11/19 in 94 ABOi LDKT (including 14 with preformed Donor Specific Antibodies, pDSAs), 27 LDKT ABO compatible (ABOc) with pDSAs, and 21 ABOc without pDSAs) during the first five years post transplantation. During the first 5 years post-transplantation, a progression of chronic lesions (patients with a ci >0 raised from 11% to 65%, *p*<0.0001, patients with a ct >0 raised from 29% to 78%, *p*<0.0001) was observed in ABOi LDKT without pDSAs. Histological patterns of evolution were comparable to those observed in ABOc kidney transplant patients. Microvascular inflammation was lower in ABOi LDKT without pDSAs compared to those with pDSAs (ABOi or ABOc). At last follow-up, 28 months, IQR (15-48) post-transplantation, 29 patients (36%) had a severe graft dysfunction (defined by a CKD-epi eGFR < 30 mL/min/1.73m²). The donor age was a predictive factor for the development of severe kidney allograft dysfunction at last follow-up (HR= 1.05, 95% CI [1.05-1.10], p= 0.03).

Hence, long-term histological analysis of ABOi LDKT shows only an increase of chronic interstitial and tubular atrophy changes, without active lesions. These data confirm that ABOi LDKT programs can be securely developed.

## Introduction

ABO incompatible (ABOi) living donor kidney transplantation (LDKT) was developed to overcome organ shortage. Although ABOi transplantation is considered to be a safe alternative to ABO compatible (ABOc) kidney transplantation (1, 2), it is associated to a higher mortality in the early period post transplantation, and a lower graft survival at one year (1, 3). While the over-mortality rate after ABOi LDKT was attributed to increased severe infection rates, the higher risk of early graft loss was associated to higher risks of surgical complications (such as surgical site bleedings), acute rejection, and polyomavirus associated nephropathy (PVAN) (4–8). Moreover, recent large meta-analyses suggested a poorer long-term patients’ and grafts’ outcomes after ABOi LDKT compared to ABOc LDKT (3, 9).

Protocol biopsies performed during the first year post transplantation, showed similar results in ABOi and ABOc LDKT (10–15). However, little is known about the long-term histology in ABOi kidney transplantation. In the present study, we describe the histological findings observed up to five years after ABOi living donors kidney transplantation transplanted and followed in our center (Nephrology and Organ transplant Department, CHU de Toulouse, France). We compared these histological features to those observed in a group of ABOc LDKT patients with preformed donor specific antibodies (DSAs) and to another group of ABOc kidney-transplant patients without DSAs. Finally, we aimed to compare the observed death-censored graft survival after one year with that was predicted by the iBox score system (an allograft survival prediction system).

## Patients and methods

Between March 2011 and November 2019, 94 ABOi LDKT (including 14 with preformed DSAs) and 41 ABOc LDKT with preformed DSAs were performed in our institution (Nephrology and Organ transplant Department, CHU de Toulouse, France). ABO compatible kidney transplant patients without DSAs didn’t undergo protocol biopsies in our institution except those grafted for IgA nephropathy between 2010-2014. During this interval, 21 patients were transplanted (6 from living and 15 for deceased donors) and histological findings were compared to ABOi and ABOc with preformed DSAs LDKT. Patients’ characteristics are presented in **Table 1**.

**Table 1:** Main patients’ characteristics.

	ABOi DSA-(n = 80)	ABOi DSA+(n = 14)	ABOc DSA-(n = 21)	ABOc DSA+(n = 27)
**Recipient**				
Age (yr), mean (± SD)	47.7 (± 13.5)	49.6 ( ± 16.1)	46.1 (± 12.4)	49.9 ( ± 11.9)
Male (%)	57 (71.3) *	5 (35.7) *	12 (57.1) *	7 (25.9) *
Graft number ≥ 2, n (%)	12 (15.0) **	8 (57.1) **	0 (0)	16 (59.3)
Preemptive KT, n (%)	35 (43.8) ***	2 (14.3) ***	5 (23.8)	8 (29.6)
Diabetes (%)	11 (13.8)	1 (7.1)	0 (0)	4 (14.8)
Primary disease, n (%)		****	
PKD IgA nephropathy Diabetes Nephroangiosclerosis Other glomerulonephritis Malformative uropathy Other	14 (17.5) 12 (15.0) 8 (10.0) 8 (10.0) 13 (16.3) 6 (7.5) 19 (23.8)	0 (0) 0 (0) 1 (7.1) 0 (0) 4 (28.6) 5 (35.7) 4 (28.6)	**0 (0)** **21 (100)** **0 (0)** **0 (0)** **0 (0)** **0 (0)** **0 (0)**	4 (14.8) 6 (22.2) 2 (7.4) 3 (11.1) 7 (25.9) 1 (3.7) 4 (14.8)
Initial IsoAgglutinine titer median (IQR), IgG				
IgM Initial anti-HLA DSA MFI, mean ± SD	64 (16; 128) 32 (8; 64) -	128 (80; 224) 20 (16; 64) 9400 ± 4900	- -	- - 8500 ± 5700
Immunosuppression induction Polyclonal antibodies (%) Basiliximab (%)	***** **53 (66.3)** **27 (33.8)**	***** **14 (100)** **0 (0)**	***** **9 (45.0)** **11 (55.0)**	***** **27 (100)** **0 (0)**
**Donor**				
Age (yr), mean (± SD)	51.2 (± 11.2)	51.2 ( ± 13.7)	51.8 ( ± 16.0)	53.4 ( ± 12.0)
Male (%)	27 (33.8)	7 (50)	12 (57.1)	9 (33.3)
Diabetes (%)	0 (0)	0 (0)	0 (0)	0 (0)
Cold ischemia (min), mean (± SD)	248.4 (± 67.2)	277.2 ( ± 108.4)	577.6 ( ± 268.8)	252.9 ( ± 50.9)
Warm ischemia (min), mean (± SD)	60.2 (± 18.4)	65.9 ( ± 32.7)	50.7 ( ± 19.4)	60.8 ( ± 29.1)

DSA, Donor-Specific Alloantibodies; ABOi, ABO incompatible; ABOc, ABO compatible; yr, year; KT, kidney transplantation; PKD, Polycystic Kidney Disease; ATG/ALG,

*ABOi DSA- vs ABOi DSA+ : p= 0.01; ABOi DSA- vs ABOc DSA- p<0.0001; ABOi DSA- vs ABOc DSA+ p<0.0001.

**ABOi DSA- vs ABOi DSA+ : p= 0.001.

***ABOi DSA- vs ABOi DSA+ : p= 0.04.

****ABOc DSA- vs ABOi DSA- p<0.0001, ABOc DSA- vs ABOi DSA+ : p<0.0001, ABOc DSA+ : p<0.0001.

*****ABOi DSA- vs ABOi DSA+ : p= 0.01; ABOi vs ABOc DSA+ : p<0.0001; ABOi DSA+ vs ABOc DSA+ : p= 0.0006; ABOi DSA+ vs ABOc DSA- p<0.0001; ABOc DSA+ vs ABOc DSA- p= 0.001.

Bold values are statistically different in the different groups.

According to French law (Loi Jardé), anonymous retrospective studies do not require Institutional Review Board approval. However, as recommended it was registered in the register of retrospective studies of the Toulouse University Hospital (RnIPH 2021-94) and covered by the MR-004 (CNIL number: 2206723 v 0), (**Supporting Document 1**).

For ABOi LDKT, recipients had undergone an apheresis (immunoadsorption or double filtration plasmapheresis) to obtain a pre-operative IgG titer <1/8. In addition, they were given rituximab (375 mg/m^2^ once for ABOi without preformed DSA and twice for those with preformed DSAs). ABOc LDKT with preformed DSAs were also treated with apharesis and 2 infusions of rituximab. Patients with preformed DSAs were all given induction therapy with polyclonal antibodies while others received either polyclonal antibodies or basiliximab. Maintenance immunosuppression was started 15 days before transplantation and included tacrolimus (target trough level 10-12 g/mL during the first 3 months post-transplantation, and 8-10 ng/mL thereafter), mycophenolic sodium (Myfortic^®^ 720 mg b.i.d until day 15 post-transplantation) and steroids. Since 2017, a switch from mycophenolic sodium to everolimus (Certican^®^ 1.5 mg b.i.d) was initiated after 15 days post transplantation. Patients from the control group who were grafted for IgA nephropathy were also given tacrolimus, mycophenolic acid and steroids. Valgancyclovir prophylaxis was given according to donor/recipient status and type of induction therapy for 3 or 6 months. All patients were given cotrimoxazole prophylaxis for 1 year.

### Immunological analyses

All patients were screened for *de novo* DSAs at month 3, and 12 post-transplantation, and thereafter every year. The presence of preformed or *de novo* DSAs was assessed by Luminex assays. Luminex assays determined the specificity of class I HLAs in A/B and class II in DR/DQ IgG antibodies in the recipients’ sera (centrifuged at 10,000*g* for 10 min) using Labscreen single Ag HLA class-I and class-II detection tests (One Lambda, Canoga Park, CA), according to the manufacturer’s instructions. The presence and specificity of antibodies were then detected using a Labscan 100^®^, and the mean fluorescence (baseline) value for each sample in each bead was evaluated. The baseline value was calculated as follows: (raw sample mean fluorescence intensity [MFI] − raw negative serum control MFI) – (negative-bead raw MFI sample − negative-bead raw MFI negative serum control). A baseline value of >1000 was considered positive.

Isoagglutinins were detected with a tube hemagglutination technique, according to the previously described technique (16).

### Histological analyses

In the present study, only protocol biopsies were analyzed, and reviewed by two pathologists specialized in kidney transplantation. For ABOi patients, these were scheduled at months 1 and 6, and thereafter at 1, 2, and 5 years post-transplantation. In ABOi LDKT patients without preformed DSAs (n=80), 226 biopsies were performed. The median number of biopsies/patient was 3 (IQR 2; 4). Sixty-one (76.3%), 52 (65.0%), 54 (67.5%), 41 (51.3%), and 18 (22.5%) patients had undergone a protocol biopsy at months 1, 6, and 1, 2 and 5 years, respectively (**Supporting Document 2**). The others were not done either due to medical reasons or patients’ refusal.

For ABOc patients with preformed DSAs, protocol biopsies were scheduled at month 6 and 1, 2, and 5 years. 27 out of 41 patients had at least one interpretable kidney biopsy during follow up. For ABOc patients without DSA grafted for IgA nephropathy, protocol biopsies were performed at 1 month, 1 year and 5 years post-transplant. Seven (33.3%), 7 (33.3%), and 16 (76.2%) patients had undergone a protocol biopsy that fitted to the standard analyses criteria at months 1, 1 year and 5 years post-transplantation, respectively. Histological findings were classified by our local, transplant renal pathologist according to the Banff 2017 classification (17). C4d staining was done by immunofluorescence technique and was scored, as positive or negative.

### Allograft loss risk prediction using iBox scores

We aimed to compare the observed graft survival after one year and that was predicted by the iBox score system, an allograft survival prediction system. The observed death-censored graft survival was assessed at 3, 5 and 7 years post transplantation. Individual allograft survival probabilities were determined using the iBox scoring system (18). Data from patients completing the 1-year visit including a kidney biopsy were included for generation of iBox risk-prediction scores. The iBox scores were generated using eight parameters: time from transplant to biopsy, eGFR and urine protein/creatinine ratio at biopsy, presence of circulating DSA, and histological findings (glomerulitis, peritubular capillaritis, interstitial inflammation, tubulitis, transplant glomerulopathy, and interstitial fibrosis/tubular atrophy) classified according to Banff score.

### Statistical analyses

Reported values represent the means (± SD) or medians (ranges). Quantitative variables were compared using the Mann–Whitney, or Kruskal-Wallis (if appropriated) non-parametric test. Categorical variables are expressed as percentages and compared between groups using the chi-squared tests or if appropriated the Fisher’s exact test. Cumulative probability of survival was calculated using the Kaplan-Meier method. Severe allograft dysfunction was defined by an estimated CKD-EPI glomerular filtration rate lower than 30 mL/min/1.73m². A cox-regression analysis was performed to identify predictive factors for graft survival without severe dysfunction (defined by an estimated CKD-EPI glomerular filtration rate lower than 30 mL/min/1.73m²). Variables with a *p*-value <0.2 in the univariate analysis were included into stepwise multivariate models. Statistical analyses were performed using the graphPad Prism 7 (San Diego, CA, USA) and Xlstat softwares (Addisoft, Paris, France).

## Results

### Description of histological lesions during the first five years post transplantation in ABOi LDKT patients without preformed DSAs

**Figure 1:**
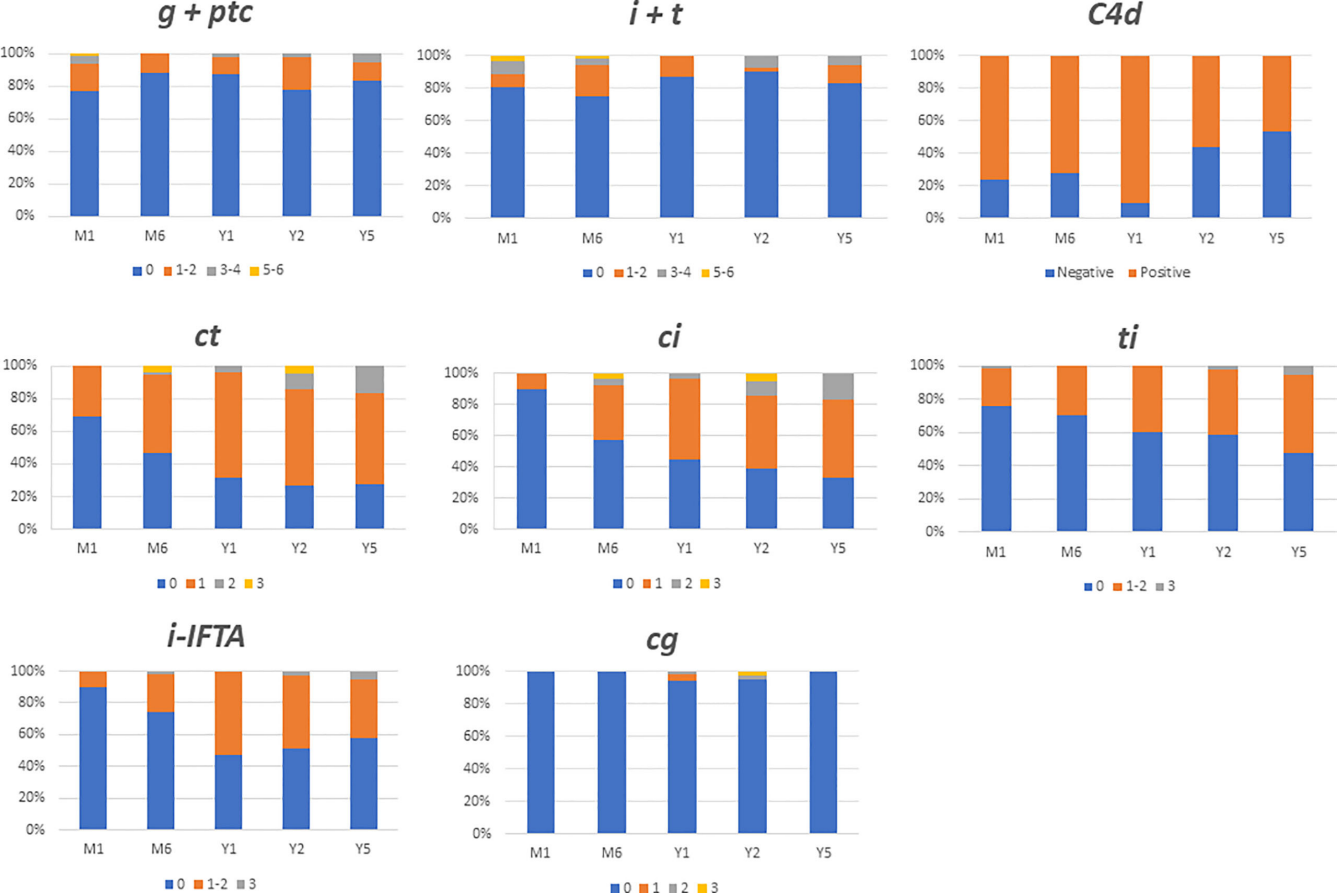
Main histological findings during 5 years follow up after ABO incompatible kidney transplantation. Data are expressed in each colomn with the percentage of patients presenting the same banff score. i, interstitial inflammation; t, tubulitis; g, glomerulitis; ptc, peritubular capilaritis; ci, chronic interstial fibrosis; ct, tubular atrophy; cg, glomerular basement membrane double coutours.

The outcome of histological lesions is presented in **Figure 1** and **Table 2**. Overall, interstitial fibrosis score (ci), and tubular atrophy (ct) increased overtime. Sixty-five out the of 80 patients (81%) had at least two interpretable kidney biopsies during the follow-up. The median times between transplantation and the first analyzed biopsy and between the first and last biopsy were 1 IQR (1;1) months and 23 IQR (11; 59) months, respectively. Between the first and last analyzed biopsy, an increase of ci (patients with a ci >0 raised from 11% to 65%, p<0.0001), ct (patients with a ct >0 raised from 29% to 78%, p<0.0001), and i-IFTA scores (patients with i-IFTA>0 raised from 31% to 54%, p= 0.01) was observed. To note, three patients with a diagnosis of PVAN that received a follow-up biopsy post diagnosis presented a ci>0 and ct>0. Patients with i-IFTA scores > 0 at last biopsy, presented lower graft function at last follow-up in comparison with patients with a i-IFTA score =0 or patients that did not present IFTA (**Supplementary Document 3**). Only 6 patients had an i-IFTA score > 2 at last biopsy: 3 had PVAN, 1 had recurrent IgA nephropathy and 2 patients had chronic active TCMR. Chronic AMR was observed in 3 patients (4.6%) at first biopsy and 6 (9.2%) at last biopsy (*p*= 0.49). No occurrence of acute AMR or acute TCMR rejection was observed at last biopsy.

**Table 2:** Results of follow-up biopsies during the 5 years post transplantation after ABO incompatible kidney transplantation without preformed DSA.

	Biopsy M1 (n = 61)	Biopsy M6 (n = 52)	Biopsy Y1 (n = 54)	Biopsy Y2 (n = 41)	Biopsy Y5 (n = 18)	p
**Histological findings**						0.09
i+ t	49 (80)	39 (75)	47 (87)	37 (90)	15 (83)	
0	5 (8)	10 (19)	7 (13)	1 (2)	2 (11)	
1-2	7 (12)	3 (6)	0	3 (8)	1 (6)	
≥3						0.53
g+ptc	47 (77)	46 (88)	47 (87)	32 (78)	15 (83)	
0	10 (16)	6 (12)	6 (11)	8 (20)	2 (11)	
1-2	4 (7)	0	1 (2)	1 (2)	1 (5)	
≥3						0.32
V	61 (100)	50 (96)	54 (100)	40 (98)	18 (100)	
0	0	2 (4)	0	1 (2)	0	
>0						**<0.0001**
Ci	55 (90)	30 (58)	24 (44)	16 (39)	6 (33)	
0	6 (10)	20 (38)	30 (56)	23 (56)	12 (66)	
1-2	0	2 (4)	0	2 (5)	0	
≥3						**0.0001**
Ct	42 (69)	25 (48)	17 (31)	11 (27)	5 (28)	
0	19 (31)	25 (48)	37 (69)	28 (68)	13 (72)	
1-2	0	2 (4)	0	2 (5)	0	
≥3						0.65
Cv	44 (72)	38 (73)	33 (61)	27 (66)	13 (72)	
0	17 (28)	14 (27)	21 (39)	14 (34)	5 (28)	
>0						0.10
Cg	61 (100)	61 (100)	51 (94)	39 (95)	18 (100)	
0	0	0	3 (6)	2 (5)	0	
>0						0.97
aah	50 (82)	44 (85)	45 (83)	34 (83)	16 (89)	
0	11 (18)	8 (15)	9 (17)	7 (17)	2 (11)	
>0						0.34
mm	56 (92)	47 (90)	43 (80)	35 (85)	16 (89)	
0	5 (8)	5 (10)	11 (20)	6 (15)	2 (11)	
>0						0.14
ti	47 (77)	37 (71)	33 (61)	25 (61)	8 (44)	
0	13 (21)	15 (29)	21 (39)	15 (37)	9 (50)	
1-2	1 (2)	0	0	1 (2)	1 (6)	
≥3						**<0.0001**
i-IFTA	56 (92)	39 (75)	27 (50)	22 (54)	10 (56)	
0	5 (8)	13 (25)	27 (50)	19 (46)	8 (44)	
>0	39/55 (69.6)	134/50 (65.4)	46/51 (85.2)	23/41 (56.1)	7/15 (46.7)	**0.001**
C4d positivity (%)						

Statistical analyses were performed using a chi-square test or Fisher’s exact test when appropriate.

i, interstitial inflammation; t, tubulitis; g, glomerulitis; ptc, peritubular capilaritis; v, vasculitis; ci, chronic interstial fibrosis; ct, tubular atrophy; cv, vascular fibrous intimal thickening; cg, glomerular basement membrane double coutours; mm, mesangial matrix expansion; aah, hyaline arteriolar thickening; ti, total inflammation; i-IFTA, inflammation in area of interstitial fibrosis/tubular atrophy.

Bold values are statistically different in the different groups.

In the 18 ABOi LDKT patients who had undergone kidney biopsies at month 1 and 5 years, there were also an increase in interstitial fibrosis (patients with a ci >0 raised from 22% to 67%) and tubular atrophy (patients with a ct >0 raised from 39% to 72%).

### Comparison of histological lesions between ABOi LDKT and ABOc patients without preformed DSAs

**Figure 2:**
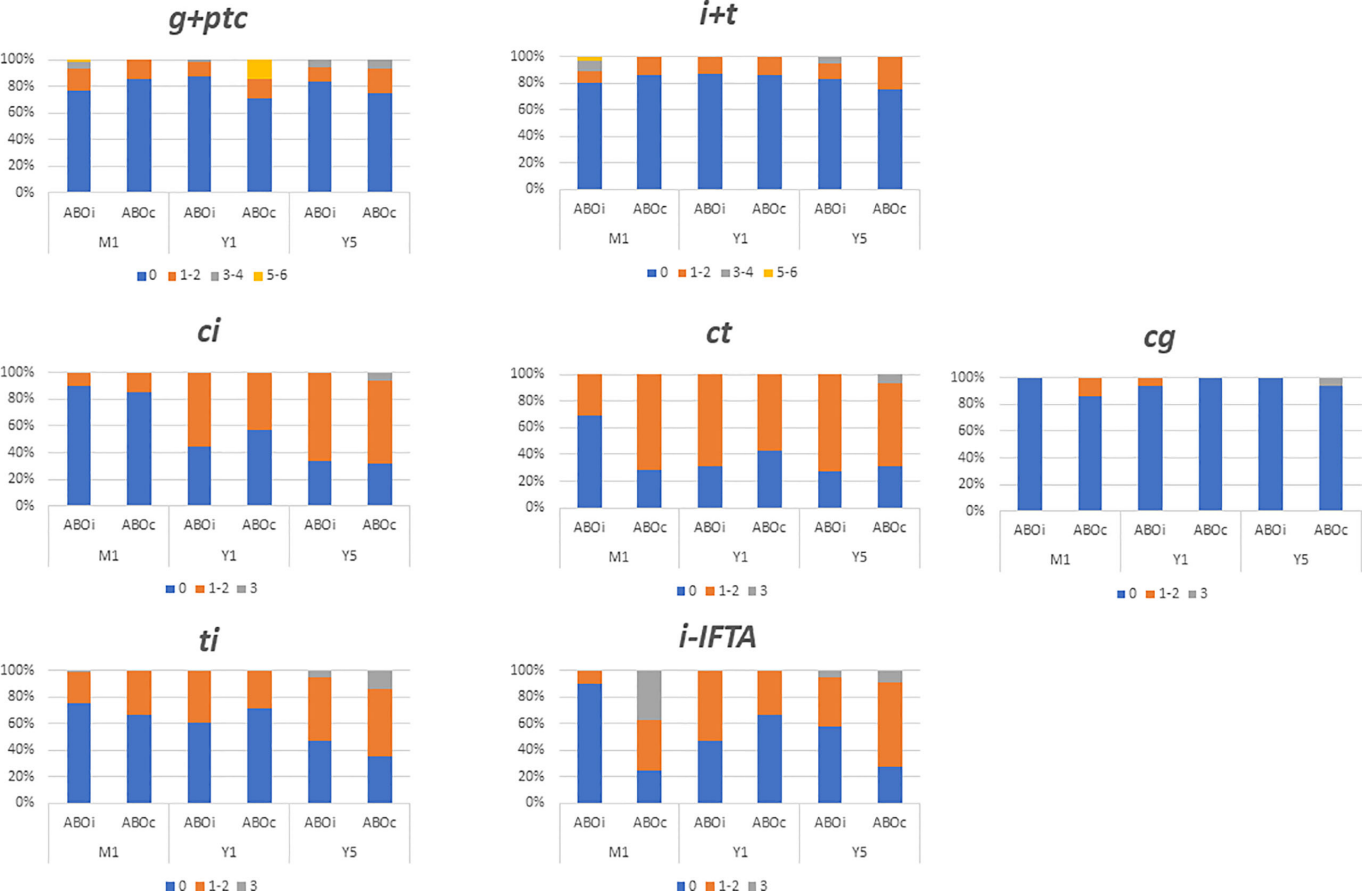
Comparison of biopsy finding at month one (M1), year one (Y1) and years five (Y5) between ABO incompatible (ABOi) and ABO compatible (ABOc) recipients. Data are expressed in each colomn with the percentage of patients presenting the same banff score. i, interstitial inflammation; t, tubulitis; g, glomerulitis; ptc, peritubular capilaritis; ci, chronic interstial fibrosis; ct, tubular atrophy; cg, glomerular basement membrane double coutours.

The progression of interstitial fibrosis and tubular atrophy was similar between ABOi and ABOc recipients during the follow-up. At year 5 post-transplantation, 67% of ABOi and 71% of ABOc recipients presented a ci score >0, and a (*p*> 0.99) ct score >0 was noted in 72% of ABOi and 71% of ABOc recipients (*p*> 0.99), (**Figure 2**; **Supplementary Document 4**).

### Comparison of histological lesions between ABOi LDKT with and without preformed DSAs and ABOc LDKT with preformed DSAs

The microvascular inflammation remained low and stable during the follow-up in ABOi LDKT without DSAs (23%, 13%, and 17% at month 1, 12 and year 5 respectively presented a g+ptc>0) chi-square test *p*= 0.37). Conversely, at 5 years post-transplantation, micro-vascular inflammation was higher in patients with preformed DSA. A g+ptc>0 was found in 88% of ABOc with preformed DSA, 57% of ABOi with preformed DSAs, and 17% in ABOi without preformed DSA (*p*= 0.002), (**Supplementary Document 5**). Futhermore, glomerular basement double contours was significantly higher in ABOc with preformed DSAs recipients compared to ABOi without preformed DSAs (cg score >0 50% and 0% for ABOc with preformed DSA, and ABOi without preformed DSAs, *p*= 0.005).

### Kidney allograft outcome after ABOi LDKT without preformed DSAs

#### Kidney-allograft survival

During the follow-up (median time 28 months, IQR (15-48)), 9 (11.3%) patients died, and eleven (13.8%) other patients lost their grafts. The median time between transplantation and graft failure was 17 IQR (2; 26) months. The causes for graft failure were PVAN (n=4), vascular graft thrombosis during the first week (n=3), ABMR (n=2), surgical complication (n=1) and reoccurrence of initial nephropathy (n=1).

**Figure 3:**
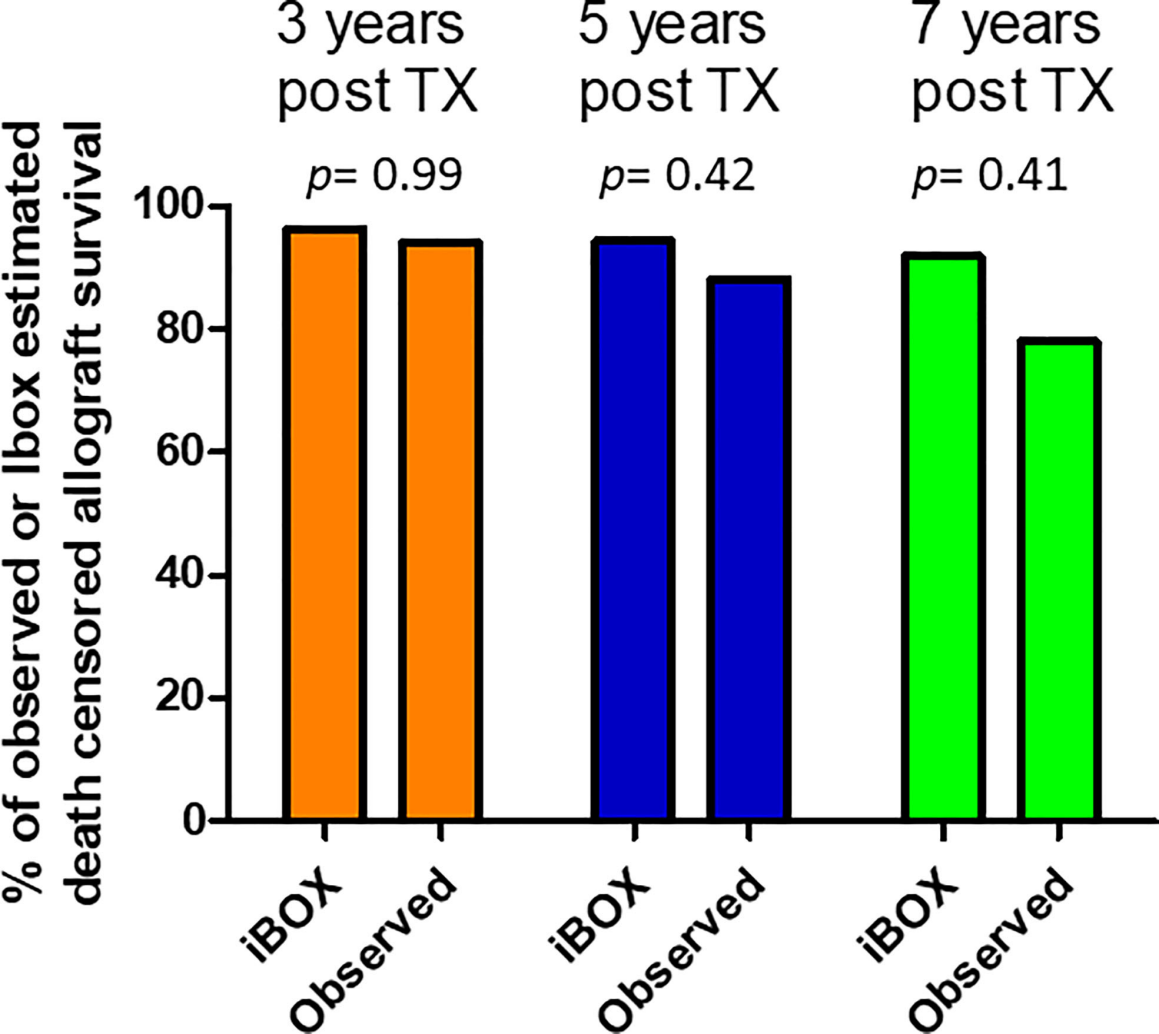
Comparison of Ibox predicted and observed death censored allograft survival at years 3, 5 and 7 post transplantation. The obtained mean iBOX score at each time post transplantation was compared with the observed graft survival.

For the 54 ABOi LDKT without preformed DSAs who had undergone a one-year allograft biopsy, we used the iBOX score to predict the death-censored graft survival. The obtained mean iBOX score at each time post transplantation was compared with the observed graft survival. The observed graft survival at 3, 5 and 7 years did not differ significantly from the predicted ones (**Figure 3**).

#### Acute rejection

During the first year, 17 patients experienced an acute rejection episode, 11 considered as TCMR and 6 ABMR. Subclinical rejection occurred in 3 patients (2 TCMR, 1 ABMR), at month 1 post transplantation. All TCMR episodes received steroid pulses. ABMR recipients received steroid pulses (n=2), plasmapheresis (n=5), eculizumab (n=3), or rituximab infusion (n=1). No acute rejection was observed beyond one year. None developed a *de novo* DSA.

#### Kidney function

Kidney function at 1 year was at 54.2 ± 21.0 ml/min/1.73m². At last follow-up, 28 months, IQR (15-48) post-transplantation, 29 patients (36%) were considered to have a severe graft dysfunction defined by a CKD-EPI eGFR lower than 30 mL/min/1.73m² (**Table 3**; **Supporting Document 6A**). In univariate analysis, the presence of delayed graft function and the donor age were associated with a severe kidney allograft dysfunction at last follow-up (Delayed graft function: 27.6% vs 7.8% in patients presenting or not a severe kidney allograft dysfunction at last follow-up, p=0.02; donor age: 55.2 ± 7.2 years, vs 48.7 ± 12.3 years in patients presenting or not a severe kidney allograft dysfunction at last follow-up, p= 0.008). After multivariate analysis, donor age (coded as a continuous variable) remained a slight predictive factor for the development of severe graft dysfunction after ABOi LDKT (HR= 1.05, 95% CI [1.05-1.10], p= 0.03). Interestingly, neither an history of acute rejection nor the pre-desensitization isoagglutinin titer, nor initial or follow-up histological lesions were associated with severe graft dysfunction.

**Table 3:** Comparison of ABOi recipients without DSA according to the development of severe graft dysfunction (defined by a CKD-Epi eGFR<30 mL/min/1.73m²) at last follow-up.

variable	Severe graft dysfunction (n = 29)	No severe graft dysfunction (n = 51)	*p*-value
**Delayed graft function, yes (%)**	**8 (27.6)**	**4 (7.8)**	**0.02**
PVAN yes n(%)	6 (20.7)	4 (7.8)	0.10
Past of rejection, yes n(%)	6 (20.7)	11 (21.6)	>0.99
isoagglutinin M titer at D0, median IQR	2 (1; 4)	2 (1; 2.5)	0.56
isoagglutinin G titer at D0, median IQR	4 (2; 32)	4 (2; 8)	0.85
Initial isoagglutinin M titer, median IQR	32 (16; 128)	16 (8; 64)	0.24
Initial isoagglutinin G titer, median, IQR	64 (16; 320)	64 (20; 224)	0.70
HLA class I and II mismatches scores	5.0 ± 2.3	5.0 ± 2.2	0.94
Warm Ischemia Time (min), min ± SD	61 ± 19	60 ± 18	0.65
Cold Ischemia Time (min), min ± SD	235 ± 50	256 ± 70	0.40
Time of dialysis before Tx (months, median, IQR)	12.5 (4.5; 32.5)	10 (0.0; 19.0)	0.24
Recipient gender, male, n (%)	18 (62)	37 (73)	0.79
Recipient age, years (mean ± SD)	48.5 ± 15.1	47.3 ± 12.7	0.70
Donor gender, male, n (%)	8 (28)	19 (37)	0.53
**Donor age, years (mean ± SD)**	**55.2 ± 7.2**	**48.7 ± 12.3**	**0.008**
Post-Tx 1 month biopsy*			
i+ t >0 (%) g+ptc >0 ci+ ct>0 i-IFTA >0 C4d positivity	5 (28) 7 (39) 6 (33) 1 (5) 11 (65)	7 (16) 7 (16) 13 (30) 4 (9) 31 (82) 1.0 (0.5; 1.0)	0.56
Post-Tx 6 months biopsy **			
i+ t >0 g+ptc >0 ci+ct>0 i-IFTA C4d positivity	5 (31)3 (19) 12 (75) 5 (31) 10 (67)	8 (22) 3 (8) 19 (53) 8 (22) 26 (35)	
Post-Tx 1Y biopsy***			
i+ t >0 g+ptc >0 ci+ct>0 i-IFTA C4d positivity	3 (19) 2 (13) 13 (81) 9 (56) 14 (93)	4 (11) 5 (13) 25 (69) 18 (47) 32 (89)	
Post-Tx 5Y biopsy****			
i+ t >0 g+ptc >0 ci+ct>0 i-IFTA C4d positivity	1 (20) 1 (20) 5 (100) 3 (60) 1 (25)	2 (15) 2 (15) 8 (62) 5 (38) 6 (50)	

PVAN, PoliomaVirus Associated Nephropathy; Tx, Transplantation; i, interstitial inflammation; t, tubulitis; g, glomerulitis; ptc, peritubular capilaritis; v, vasculitis; ci, chronic interstial fibrosis; ct, tubular atrophy; cv, vascular fibrous intimal thickening; cg, glomerular basement membrane double coutours; mm, mesangial matrix expansion; aah, hyaline arteriolar thickening; ti, total inflammation; i-IFTA, inflammation in area of interstitial fibrosis/tubular atrophy.

*Results present here concerned 15 patients with severe graft dysfunction, and 39 without.

**Results present here concerned 15 patients with severe graft dysfunction, and 33 without.

***Results present here concerned 16 patients with severe graft dysfunction, and 44 without.

****Results present here concerned 5 patients with severe graft dysfunction, and 13 without.

Bold values are statistically different in the different groups.

To note, while the urine albumin/creatinine ratio remained stable in ABOi LDKT without preformed DSAs during the first five years post transplantation (median urine albumin/creatinine ratio: from 11.0 mg/g, IQR (0.0; 60.0) at one year to 26.0 mg/g, IQR (3.0; 100.0) at five years, p=0.28), an increase of proteinuria was observed in ABOi LDKT with preformed DSAs (from 0.0 mg/g, IQR (0.0; 0.0) at one year to 1000.0 mg/g, IQR (2.5; 2000.0) at five years, p=0.004), (**Supplementary Document 6B**).

## Discussion

Herein, we assessed the long-term histological pattern after ABOi LDKT. Overall, our study showed few histological modifications. During the five years post-transplantation, only chronic lesions (chronic interstitial fibrosis, “ci”, and chronic tubular atrophy, “ct”) worsened in ABOi LDKT. Similar results were observed by Masutani and colleagues who performed protocol biopsies at three and 12 months post-transplantation in 226 ABO compatible without preformed DSAs and 101 ABOi LDKT (10). In a retrospective study performed between 1999 and 2006, Bentall and collegues compared histological patterns at one- and five year post-transplant between LDKT patients with preformed DSAs (Positive crossmatches, n=102), ABOi without preformed DSAs (n=73), and 652 ABOc patients (19). They found higher rates of chronic injuries and inflammation in patients with positive crossmatch at transplantation compared to ABOi and ABOc transplants. Nonetheless, they also observed higher rates of chronic histological changes such as chronic glomerulopathy in ABOi group comparing with ABOc LDKT. However, this later study was done before the era of sensitive antibodies detection methods, modern immunosuppression, and Banff classification. This prompted us to investigate the long-term histological outcome post ABOi transplantation. For this purpose, we compared histological lesions in a group of ABOi LDKT and a group of ABOc without preformed DSAs patients transplanted for an IgA nephropathy. Interestingly, we did not observe any difference between both groups at 5 years after transplantation. We did not observe active microvascular inflammation in protocol biopsies. The occurrence of acute antibody mediated rejection is a classical and severe complication of the early transplant period after ABOi transplantation (20), possibly due to a hemagglutination assays limitations (21, 22) or complement control limitation (23). After this period, mechanisms of accommodation develop, preventing long-term acute and chronic rejection. Tasaki and colleagues demonstrated that the production of donor-specific blood group antibodies was downregulated in patients with stable graft function (24). ABO carbohydrate antigen recognition is a T-cell independent immunological response, in which innate B cells are considered to be the source of Isoagglutinines (2). However, these cells possess intrinsic regulatory function through the release of IL-10, depending on the site and environment (25). It was previously observed higher blood level of TGFβ and IL-10 in ABOi stable patients comparing with ABOc transplantation (24, 26). Others mechanisms are suspected such as intra-graft complement regulation could also participate (23). The absence of microvascular inflammation observed five years after transplantation in our study reinforces the role of accommodation in this setting. Moreover, the lack of development of transplant vasculopathy observed in our study could be explained by the absence of expression of ABO antigens by kidney smooth muscular cells (2). This is in starck contrast with transplantation with anti-HLA donor specific antibodies where chronic rejection remains a major cause of graft loss (27). As expected, in our study we found a progression of microvascular inflammation, and chronic rejection after transplantation with preformed DSA, regardless of the ABO compatibility. These results confirm the efficacy of current desensitization strategies and maintenance immunosuppression to overcome the ABO barrier.

In ABOi LDKT without preformed DSAs, we observed an increase of i-IFTA scores during the follow-up. As expected (28), at last follow-up, a lower glomerular filtration rates was observed in patients with i-IFTA compared to those without i-IFTA. Isolated i-IFTA is not specific lesion and can be observed in case of chronic TCMR, ABMR and poliomavirus-associated nephropathy.

Conflicting results were previously published concerning the long term kidney function after ABO incompatible kidney transplantation, comparing with ABO compatible transplantation (3, 9, 29, 30). However, early patient death from infection related to desensitization, and early antibody mediated rejection are responsible for the observed difference concerning graft survival. Similar results after one year post transplantation with ABO compatible LDKT is consistent with the comparable histological changes observed in our study between ABOi and ABOc patients (30).

This study has several limitations. First, this is a single-center study with small sample. However, even if some adjustments were made (with the use of mTOR inhibitors in place of MMF/MPA), patients had comparable maintenance immunosuppression strategy and histological pattern was re-analyzed by two specialized pathologists and classified according to the Banff classification. Second, to assess the histological changes after ABO incompatible transplantation, we compared kidney biopsies to those obtained in a group of patients transplanted for IgA nephropathy that had undergone protocol biopsies. We cannot exclude an overestimation of chronic lesions in our control group due to the reoccurrence of initial glomerular disease. Nevertheless, it has been shown that five-years histology and graft survival did not differ between patients grafted for IgA nephropathy and those transplanted for other reasons (31). In addition, we didn’t observe active lesions between ABOc and ABO-incompatible LDKT.

In conclusion, long-term histological analysis of ABO incompatible kidney transplantation shows only an increase of chronic interstitial and tubular atrophy changes, without active lesions. Histological pattern is comparable to the one observed in ABO-compatible kidney transplant recipients. These data corroborate the finding that ABOi LDKT programs can be securely developed.

## Data availability statement

The original contributions presented in the study are included in the article/**Supplementary Material**. Further inquiries can be directed to the corresponding author.

## Ethics statement

The studies involving human participants were reviewed and approved by Toulouse University Hospital (RnIPH 2021-94). Written informed consent for participation was not required for this study in accordance with the national legislation and the institutional requirements.

## Author contributions

Each author mentioned have participated in the work as follow: AB, NK, and PG designed the study, analyzed and interpreted the data, and wrote the paper. MR analyzed the data, revised the manuscript and provided intellectual content of critical importance. OC, LE, AD, MC, NC-J revised the manuscript, and provided intellectual content of critical importance. All authors contributed to the article and approved the submitted version.

## Acknowledgments

We sincerely thank all nurses involved in our department.

## Conflict of interest

The authors declare that the research was conducted in the absence of any commercial or financial relationships that could be construed as a potential conflict of interest.

## Publisher’s note

All claims expressed in this article are solely those of the authors and do not necessarily represent those of their affiliated organizations, or those of the publisher, the editors and the reviewers. Any product that may be evaluated in this article, or claim that may be made by its manufacturer, is not guaranteed or endorsed by the publisher.

## Abbreviations

ABOi, ABO incompatible; ABOc, ABO compatible; DSAs, Donor-Specific Antibodies; LDKT, Living Donor Kidney Transplant Recipients; PVAN, PoliomaVirus Associated Nephropathy; TCMR, T Cell Mediated Rejection; ABMR, Antibody Mediated Rejection.
