# Supplementary material for: Progression of histological lesions after ABO incompatible kidney transplantation
Source: Front Immunol. 2022 Oct 6;13:969998. doi: 10.3389/fimmu.2022.969998 (PMC9582152; doi:10.3389/fimmu.2022.969998)
Supplement: Supplementary file 1 [file DataSheet_1.docx]

Supplementary documents

1. Study Registration
2. Study flowchart.
3. Comparison of ABOi recipients without DSA that underwent at least two interpretable biopsies, according to the i-IFTA status at last follow-up biopsy.
4. Comparison of biopsy findings at month one (M1), year one (Y1) and years five (Y5) between ABO incompatible (ABOi) and ABO compatible (ABOc) recipients.
5. Comparison of histological findings at one year between ABOi without preformed DSA, ABOi with preformed DSA. ABOc without preformed DSA and ABOc with preformed DSA.
6. Comparison of kidney function during the 5 years post transplantation between ABOi without preformed DSA (ABOi DSA-, n=80), ABOi with preformed DSA (ABOi DSA+, n=14).

**Supplementary document 1**

A *retrospective* analyzes of the data of ABO incompatible, living-donor, kidney transplant patients over the years 2011-2019 was performed in the Toulouse university-hospital.

According to French law on ethics, patients were informed that their codified data will be used for the study.

According to the French ethic and regulatory law (public health code) *retrospective* studies based on the exploitation of usual care data don’t should be submit at an ethic committee but they have to be declare or cover by reference methodology of the French National Commission for Informatics *and Liberties (CNIL).*

A collection and computer processing of personal and medical date was implemented to analyze the results of the research. Toulouse University Hospital signed a commitment of compliance to the reference methodology MR-004 of the French National Commission for Informatics and Liberties (CNIL). After evaluation and *validation by the data protection officer and according to the General Data Protection* Regulation*, this study completing all the criteria, it is register in the register of retrospective study of the Toulouse University Hospital (number’s register: RnIPH 2021-94) and cover by the MR-004 (CNIL number: 2206723 v 0).

This study was approved by Toulouse University Hospital and confirm that ethic requirements were totally respected in the above report.

*Regulation (EU) 2016/679 of the European Parliament and of the Council of 27 April 2016

Supplementary document 2

1. Study Flowchart


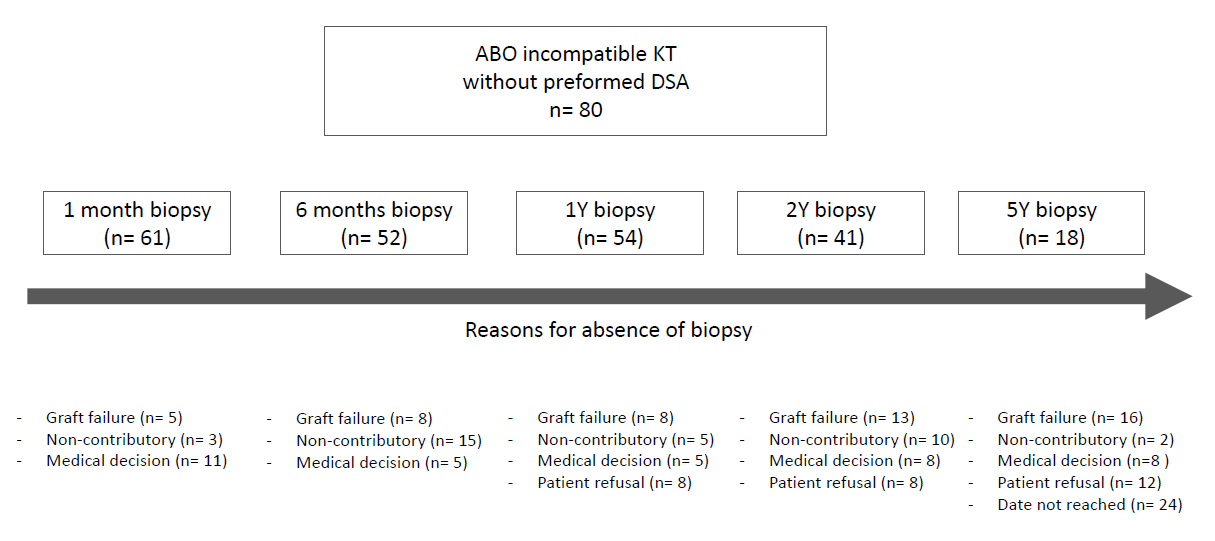


1. Number of biopsies analyzed in each group at the different timepoints

|  | M1 | M6 | Y1 | Y2 | Y5 |
| --- | --- | --- | --- | --- | --- |
| ABOi DSA – (n=80) | 61 | 52 | 54 | 41 | 18 |
| ABOi DSA+  (n=14) | 10 | 11 | 10 | 9 | 5 |
| ABOc DSA-  (n=21) | 7 | 0 | 7 | 0 | 16 |
| ABOc DSA+  (n=27) | 15 | 13 | 17 | 10 | 8 |

Abbreviations: ABOi, ABO-incompatible kidney transplantation; DSA, Donor-Specific Antibody; ABOc, ABO-compatible kidney transplantation; M, month post transplantation; Y, year post transplantation.

**Supplementary document 3. Comparison of ABOi recipients without DSA that underwent at least two interpretable biopsies, according to the i-IFTA status at last follow-up biopsy.**

| Variable | i-IFTA > 0  (n=35) | No IFTA or i-IFTA=0  (n= 30) | P |
| --- | --- | --- | --- |
| Recipient age at transplantation | 44.9 ± 2.5 | 49.7 ± 2.5 | 0.09 |
| Diabetes mellitus, y (%) | 4 (11.4) | 5 (16.7) | 0.72 |
| Previous transplantation, y (%) | 5 (14.3) | 5 (16.7) | >0.99 |
| Cold ischemia time (min), mean (± SD)  Warm ischemia time (min), mean (± SD) | 232.0 ± 8.8  59.7 ± 3.2 | 269.9 ± 14.0  60.3 ± 3.6 | 0.02  0.89 |
| Class I (A,B) and II (DR, DQ) mismatches | 4.9 ± 0.3 | 5.0 ± 0.4 | 0.74 |
| Anti-HLA (non DSA) sensitization at Tx, y (%) | 22 (62.9) | 20 (66.7) | 0.80 |
| Donor age at transplantation | 53.8 ± 1.8 | 46.5 ± 2.0 | 0.008 |
| Induction therapy   - IL-2R blockers, n (%) - Anti-lymphocyte globulins, n (%) | 15 (42.9)  20 (57.1) | 8 (26.7)  22 (73.3) | 0.20 |
| Initial isoagglutinins IgG titer, median (IQR)  Initial isoagglutinins IgM titer, median (IQR)  Isoagglutinins IgM titer at Tx, median (IQR)  Isoagglutinins IgG titer at Tx, median (IQR) | 96 (20; 256)  32 (12; 64)  4 (2; 8)  2 (1; 3.5) | 64 (14; 128)  16 (8; 64)  4 (2; 8)  2 (1; 2) | 0.47  0.60  0.67  0.14 |
| Delayed graft function, y (%) | 5 (14.3) | 4 (13.3) | >0.99 |
| Past of rejection, y (%) | 9 (25.7) | 6 (20) | 0.77 |
| Time between Tx- last-follow-up (months) | 40.3 ± 3.9 | 47.0 ± 4.8 | 0.28 |
| Time between Tx- last biopsy (months) | 29.5 ± 3.4 | 30.6 ± 3.7 | 0.83 |
| Time between last-biopsy – last follow-up (months) | 9.7 ± 2.2 | 16.2 ± 2.9 | 0.07 |
| Diagnosis of PVAN at last biopsy, yes (%) | 7 (20) | 2 (7) | 0.16 |
| Estimated CKD-Epi eGFR (mL/min/1.73m²) at last follow-up, median (IQR) | 38 (16; 54) | 59 (42; 74) | 0.0002 |

Abbreviations: i-IFTA, inflammation in area of interstitial fibrosis/tubular atrophy; DSA, Donor-Specific Alloantibodies; IL-2R, Interleukin-2 receptor; PVAN, PoliomaVirus Associated Nephropathy; CKD-Epi, Chronic Kidney Disease- EPIdemiology collaboration; eGFR, estimated Glomerular Filtration Rate.

**Supplementary document 4.** Comparison of biopsy findings at month one (M1), one year (Y1) and five years (Y5) between ABO incompatible (ABOi) and ABO compatible (ABOc) recipients without preformed DSAs.

|  | ABOi DSA- | ABOc DSA- | p |
| --- | --- | --- | --- |
| Post-Tx M1 biopsy*  g+ptc  i+t  ci  ct  cg  i-IFTA | 0.0 (0.0; 0.0)  0.0 (0.0; 0.0)  0.0 (0.0; 0.0)  0.0 (0.0 ; 1.0)  0.0 (0.0; 0.0)  0.0 (0.0; 0.0) | 1. (0.0; 0.0) 2. (0.0; 1.0)   0.0 (0.0; 0.0)  1.0 (0.0 ; 1.0)  0.0 (0.0; 0.0)  0.0 (0.0; 0.0) | 0.72  0.72  0.70  0.04  0.10  >0.99 |
| Post-Tx Y1 biopsy**  g+ptc  i+t  ci  ct  cg  i-IFTA | 0.0 (0.0; 0.0)  0.0 (0.0; 0.0)  1.0 (0.0; 1.0)  1.0 (0.0 ; 1.0)  0.0 (0.0; 0.0)  1.0 (0.0; 1.0) | 1. (0.0; 1.0) 2. (0.0; 1.0)   0.5 (0.0; 1.0)0.5 (0.0; 1.0)   1. (0.0; 0.0)   1.0 (0.25; 1.0) | 0.15  >0.99  0.67  0.27  >0.99  0.62 |
| Post-Tx Y5 biopsy***  g+ptc  i+t  ci  ct  cg  i-IFTA | 1. (0.0; 0.0) 2. (0.0; 0.0)   1 (0.0 ; 1.0)  1.0 (0.0; 1.0)  0.0 (0.0; 0.0)  0.0 (0.0; 1.0) | 1. (0.0; 0.75) 2. (0.0; 0.75)   1.0 (0.0 ; 1.0)  1.0 (0.0; 1.0)  0.0 (0.0; 0.0)  1.0 (0.0; 1.0) | 0.73  0.66  0.41  0.67  0.47  0.50 |

* Results present here concerned 61 patients with ABOi LDKT and 7 patients with ABOc KT.

** Results present here concerned 54 patients with ABOi LDKT and 7 patients with ABOc KT.

*** Results present here concerned 18 patients with ABOi LDKT and 16 patients with ABOc KT.

Abbreviations: TX, transplantation; : i, interstitial inflammation; t, tubulitis; g, glomerulitis; ptc, peritubular capilaritis; ci, chronic interstial fibrosis; ct, tubular atrophy; cg, glomerular basement membrane double coutours; ti, total inflammation; i-IFTA, inflammation in area of interstitial fibrosis/tubular atrophy.

**
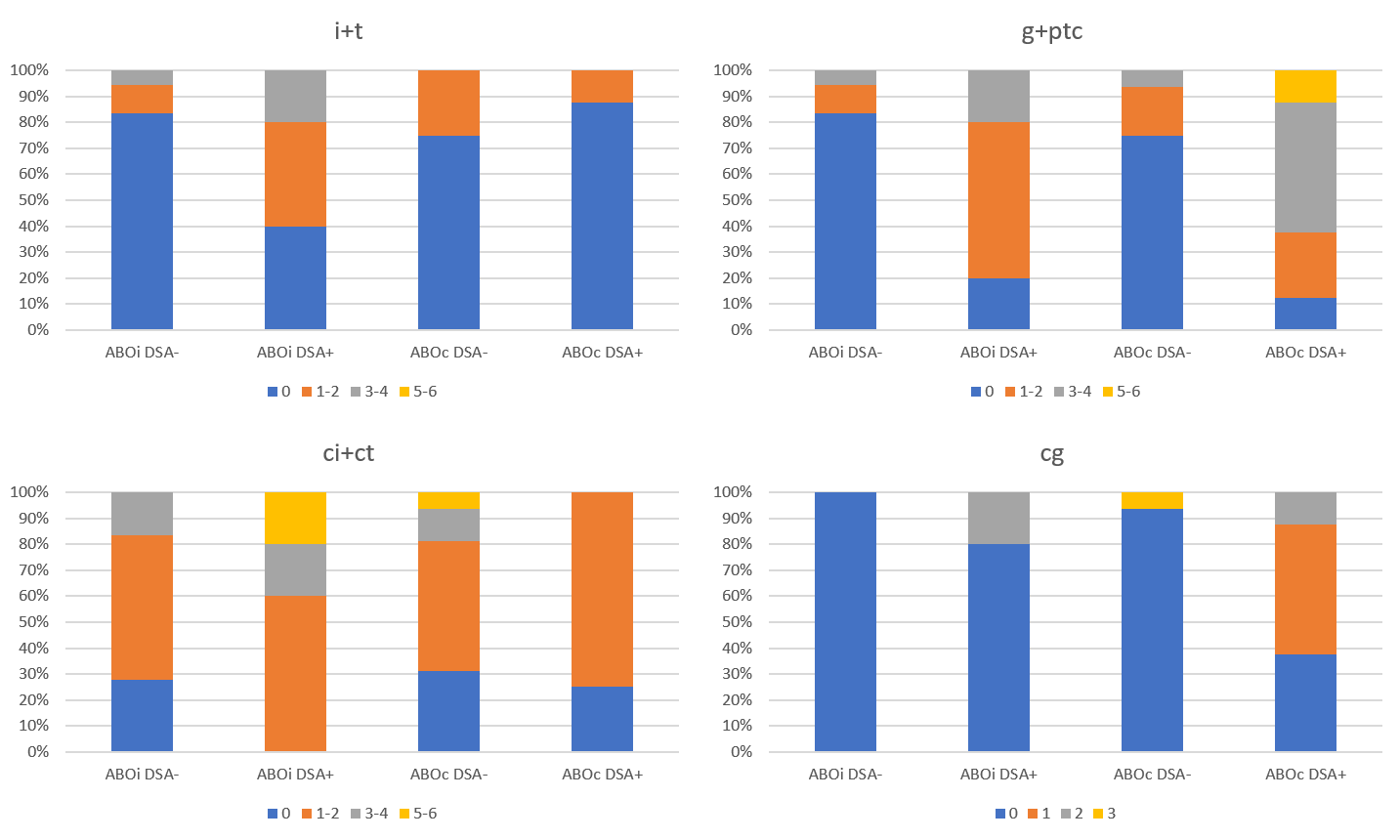
Supplementary document 5**. Comparison of histological findings at one year between ABOi without preformed DSA (ABOi DSA-, n=54), ABOi with preformed DSA (ABOi DSA+, n=10). ABOc without preformed DSA (ABOc DSA-, n=7) and ABOc with preformed DSA (ABOc DSA+, n=17) recipients. Full bars represent medians.

Abbreviations: TX, transplantation; : i, interstitial inflammation; t, tubulitis; g, glomerulitis; ptc, peritubular capilaritis; ci, chronic interstial fibrosis; ct, tubular atrophy; cg, glomerular basement membrane double coutours; ti, total inflammation.

|  | ABOi DSA- | ABOi DSA+ | ABOc DSA- | ABOc DSA+ | p |
| --- | --- | --- | --- | --- | --- |
| Post-Tx Y1 biopsy*  g+ptc  i+t  ci  ct  cg  i-IFTA | 0.0 (0.0; 0.0)  0.0 (0.0; 0.0)  1.0 (0.0; 1.0)  1.0 (0.0; 1.0)  0.0 (0.0; 0.0)  1.0 (0.0; 1.0) | 1.0 (0.0; 2.0)  0.0 (0.0; 1.0)  1.0 (0.0; 1.0)  1.0 (0.0; 1.0)  0.0 (0.0; 0.0)  1.0 (0.0; 1.0) | 0.0 (0.0; 1.0)  0.0 (0.0; 1.0)  0.0 (0.0; 1.0)  0.0 (0.0; 1.0)  0.0 (0.0; 0.0)  1.0 (0.25; 1.0) | 1.0 (0.0; 3.0)  0.0 (0.0; 1.5)  1.0 (0.0; 1.0)  1.0 (0.0; 1.0)  0.0 (0.0; 0.0)  1.0 (0.5; 1.0) | **<0.0001***  0.10  0.90  0.79  0.70  0.34 |
| Post-Tx Y5 biopsy**  g+ptc  i+t  ci  ct  cg  i-IFTA | 0.0 (0.0; 0.0)  0.0 (0.0; 0.0)  1.0 (0.0; 1.0)  1.0 (0.0; 1.0)  0.0 (0.0; 0.0)  0.0 (0.0; 1.0) | 1.0 (0.5; 2.5)  1.0 (0.0; 2.5)  1.0 (1.0; 2.5)  1.0 (1.0; 2.5)  0.0 (0.0; 1.0)  1.0 (0.5; 2.0) | 0.0 (0.0; 0.75)  0.0 (0.0; 0.75)  1.0 (0.0; 1.0)  1.0 (0.0; 1.0)  0.0 (0.0; 0.0)  1.0 (0.0; 1.0) | 3.0 (1.25; 3.75)  0.0 (0.0; 0.0)  1.0 (0.0; 1.0)  1.0 ( 0.25; 1.0)  1.0 (0.0; 1.0)  0.5 (0.0; 1.0) | **0.0004****  0.17  0.23  0.32  **0.0009*****  0.45 |

* Results present here concerned 54 patients ABOi DSA-, 10 patients ABOi DSA+, 7 patients ABOc DSA- and 17 patients ABOc DSA+. Kruskal-Wallis test was used to asses variable distribution ;

**ABOi DSA- vs ABOc DSA+ : p<0.0001** , **ABOi DSA- vs ABOi DSA+ : p=0.0009** ,

ABOi DSA+ vs ABOc DSA+ : p=0.40 , ABOi DSA- vs ABOc DSA- : p=0.11 (using Mann-Whitney test).

** Results present here concerned 18 patients ABOi DSA-, 5 patients ABOi DSA+, 16 patients ABOc DSA- and 8 patients ABOc DSA+. Kruskal-Wallis test was used to asses variable distribution ;

**ABOi DSA- vs ABOc DSA+ : p=0.0003** , ABOi DSA+ vs ABOc DSA+ : p=0.24 , **ABOc DSA- vs ABOc DSA+ : p=0.0006** , ABOi DSA- vs ABOc DSA- : p=0.73 (using Mann-Whitney test).

*** **ABOi DSA- vs ABOc DSA+ : p=0.0009** , ABOi DSA- vs ABOi DSA+ : p=0.22 , ABOi DSA- vs ABOc DSA- : p=0.47 , ABOi DSA+ vs ABOc DSA+ : p=0.33 (using Mann-Whitney test).

**Supplementary document 6**. Comparison of kidney function during the 5 years post transplantation between ABOi without preformed DSA (ABOi DSA-, n=80), ABOi with preformed DSA (ABOi DSA+, n=14).

1. CKD-Epi estimated Glomerular Filtration Rate (mL/min/1.73m²). Data are expressed as mean ± SD. A value of 0 was attributed to patients that returned to dialysis.

1. Urine albumin / creatinine ratio (in milligramme per gramme). Data are expressed as mean ± SD.
